# Bioequivalence Study of Pantoprazole Sodium-HPBCD and Conventional Pantoprazole Sodium Enteric-Coated Tablet Formulations 

**DOI:** 10.1155/2013/347457

**Published:** 2013-02-07

**Authors:** Sandesh P. Kamdi, Prashant J. Palkar

**Affiliations:** Medical Services Department, Akumentis Healthcare Ltd., 204 Second Floor, G-Corp Tech Park, Kasarvadavali, Near Hypercity, Ghodbunder Road, Thane (West), Maharashtra 400 615, India

## Abstract

The objective of this study was to investigate the bioequivalence of two formulations of 40 mg pantoprazole sodium enteric-coated tablets: Tripepsa as the test and Pantocid as the reference. The two products were administered as a single oral dose according to a randomized two-phase crossover with a 1-month washout period in 25 healthy Indian volunteers. After drug administration, serial blood samples were collected over a period of 30 hours. Plasma pantoprazole concentrations were measured by high-performance liquid chromatography with UV detection. Pharmacokinetic parameters were analyzed based on noncompartmental analysis. The logarithmically transformed data of AUC_0−*∞*_ and *C*_max_ were analyzed for 90% confidence intervals (CI) using ANOVA. The mean (90% CI) values for the ratio of AUC_0−*∞*_ and *C*_max_ values of the test product over those of the reference product were 90.21 (83.69–97.24) and 108.68 (100.21–117.86), respectively (within the bioequivalence range of 80–125%). On the basis of pharmacokinetic parameters including AUC_0−*∞*_, AUC_0−*t*_, and *C*_max_ values, both the formulations were bioequivalent.

## 1. Introduction

Pantoprazole, a proton pump inhibitor (PPI), is indicated for the treatment of erosive esophagitis associated with gastroesophageal reflux disease (GERD). Pantoprazole is one of the highly prescribed PPI in the management of peptic ulcer diseases. It shows high specificity for the relevant binding sites on activated proton pumps with little propensity to cause unwanted systemic effects [1, 2].

Pantoprazole sodium, administered as a 40 mg enteric-coated tablet, is quantitatively absorbed. Its absolute bioavailability is 77% and does not change upon multiple dosing. Following a single oral dose of 40 mg, *C*_max⁡_ is approximately 2.5 mg/L with a *T*_max⁡_ of 2-3 h. Pantoprazole is extensively metabolized in the liver and has a total serum clearance of 0.1 l/h/kg, a serum elimination half-life of about 1.1 h, and an apparent volume of distribution of 0.15 L/kg. 98% of pantoprazole is bound to serum proteins. Elimination half-life, clearance, and volume of distribution are independent of the dose. Almost 80% of an oral or intravenous dose is excreted as metabolites in urine; the remainder is found in feces and originates from biliary secretion. The clearance of pantoprazole is only slightly affected by age, with its half-life being approximately 1.25 h in the elderly [3]. Pantoprazole is an acid labile drug that requires protection from degradation in acidic media [4]. Hence, oral formulations of pantoprazole are available as enteric-coated tablets.

Cyclodextrins are nonreducing cyclic oligosaccharides, consisting of dextrose units. Cyclodextrins have a “doughnut” shape, with the interior of the molecule being relatively hydrophilic. Because of their unique chemical structure, cyclodextrins are capable of forming “inclusion complexes” with many drug molecules if the drug is capable of undergoing chemical degradation in solution. The drug molecule can be protected by inclusion complexation with a cyclodextrin [5, 6]. Amongst thousands of excipients used for modifying the physical properties of drug or for altering its biopharmaceutical characteristics, cyclodextrins can be considered as the most excellent one [7–9]. Cyclodextrins can improve the stability of a number of labile drugs against dehydration, hydrolysis, oxidation, and photodecomposition and thus increasing the shelf life of drugs. Cyclodextrin-induced enhancement of drug stability is due to inhibition of drug interaction with vehicles and/or inhibition of drug bioconversion at the absorption site. By providing molecular shield, cyclodextrin complexation encapsulates labile drug molecules at the molecular level and insulates them against various degradation processes [8, 10, 11].

Of all the cyclodextrin derivatives available, hydroxypropyl-beta-cyclodextrin (HPBCD) is the safest, as it does not permeate the membranes. A literature survey shows that the toxicity of HPBCD has been extensively studied. HPBCD has been shown to have a reduced haemolytic potential, making it suitable for parenteral use as well as for oral and/or topical applications. HPBCD encapsulation technology is well known for its solubilizing power. HPBCD is well tolerated in most species, particularly if dosed orally, and shows limited toxicity, depending upon dose and route of administration [12]. Previously an attempt has been made to evaluate the solubility of pantoprazole by using beta-cyclodextrin and HPBCD. It was found that beta-cyclodextrin and HPBD increased the solubility of pantoprazole by 4 and 36 times respectively, [13].

In the present study, a generic preparation of pantoprazole with HPBCD has been developed for clinical use. Although the generic and the innovator preparations contain the same active ingredient, they differ from each other by manufacturing processes as well as content of excipients, which affect the rate and extent of absorption of active drug. Therefore, the bioequivalence testing is mandated to confirm the bioavailability between the two preparations in human subjects. In the present study, the objective was to determine the bioequivalence of two oral formulations of pantoprazole in human subjects.

## 2. Materials and Methods

### 2.1. Subjects

Twenty-six healthy Indian male subjects aged between 20 and 32 years old and the body mass index within 18–25 participated in this study. Subjects were in good health on the basis of medical history, physical examination, and routine blood test. Subjects with known contraindication or hypersensitivity to pantoprazole were excluded as well as those with history of drug abuse, heavy alcohol consumption or cigarette smoking. No drug was allowed 1 month before the study period. The study was approved by the Research Ethics Committee in Mumbai, India.

### 2.2. Study Drugs

The reference product was commercially available 40 mg pantoprazole sodium enteric-coated tablets Pantocid manufactured by Sun Pharma, Mumbai, India (lot no. BSK0980, Mfd. 04/2011, Exp. 03/2014). Test product was formulated as 1 : 2 mixture of pantoprazole sodium with HPBCD enteric-coated tablets. Tripepsa manufactured by Akums Drugs & Pharmaceuticals Limited, India (lot no. XDCL02, Mfd. 01/2012, Exp. 12/2013).

### 2.3. Study Design and Method of Drug Administration

 The experimental design of two-way crossover and randomized study with 25 healthy male volunteers was adopted in the study. As per the randomization schedule, each subject received a single oral dose of 40 mg pantoprazole tablet (either Tripepsa or Pantocid) on the morning with 240 ± 2 mL drinking water at room temperature in sitting posture, under 10 hours overnight fasting condition. The fasting state continued for 04 hrs after dose. Water and lunch were served 2 hours and 4 hours after dose, respectively. The washout period between each treatment was 1 month. After a washout period, subjects were administered the different brand of pantoprazole in the same manner. An identical meal and fluid intake were served during the two study periods. Subjects were required to refrain from drinking caffeine containing beverages and alcohol. The blood samples for the analysis of pantoprazole in plasma and collected at the following time points. Predose blood sample (00.00 hr) was collected just after phlebotomy within 2.0 hours prior to drug administration and the postdose samples were collected at 0.30, 1.0, 1.30, 2.0, 2.20, 2.40, 3.0, 3.15, 3.30, 3.45, 4.0, 4.15, 4.30, 4.45, 5.0, 5.20, 5.40, 6.0, 7.0, 8.0, 9.0, 10.0, 12.0, 16.0, 24.0, and 30.0 hrs (each contains 1 × 5 mL) after dose, respectively. Within 30 minutes, the blood samples were centrifuged to separate the plasma. The plasma samples were immediately kept at −20°C until assay.

### 2.4. Determination of Pantoprazole Concentration in Plasma

Drug analysis of pantoprazole in plasma was performed by suitable analytical method developed and validated at the Analytical Department, Drug Monitoring Research Institute, according to the international guidelines. The assay was operated using a highperformance liquid chromatography (HPLC) with UV detector set at 288 nm. A highperformance liquid chromatography-ultraviolet detection (HPLC-UV) method was established to determine the concentration of pantoprazole in human plasma. The limit of quantification during sample analysis was concentration range for pantoprazole of 19.9 ng/mL to 5000.1 ng/mL. A Shimadzu LC-10ATvp pump (Kyoto, Japan) and a Shimadzu SPD-10Avp detector (Kyoto, Japan) were used. Chromatography was performed on a Diamonsil C18 column (particle size 5 *μ*m, 200 mm × 4.6 mm ID, Beijing, China), using a mobile phase of methanol-water (60 : 40, V/V), which was delivered at a flow rate of 1.2 mL*·*min^−1^. Under the present chromatographic conditions, HPLC retention time of pantoprazole and the IS (internal standard, betamethasone) was 6.3 min and 9.0 min, respectively. To a 500 *μ*L aliquot of plasma sample, 50 *μ*L of methanol-water (50 : 50, V/V) and 50 *μ*L of the IS solution (betamethasons 400 *μ*g*·*mL^−1^ in 50% methanol) were added. The mixed samples were then extracted with 3 mL of diethyl ether-acetic ether (3 : 2, V/V). The mixture was vortex-mixed for approximate 1 min, then shaken on a mechanical shaker for 10 min. After centrifugation at 2000 g for 5 min, the upper organic layer was removed and evaporated to dryness at 40°C under a gentle stream of nitrogen. The residue was reconstituted in 100 *μ*L of the mobile phase, then vortex-mixed. A 20 *μ*L aliquot of the resulting solution was injected onto the HPLC-UV system for analysis.

### 2.5. Statistical Methods and Data Analysis 

#### 2.5.1. Pharmacokinetic Analysis

Pharmacokinetics analysis was performed by means of a noncompartmental method. The parameters *C*_max⁡_ and *T*_max⁡_ were determined by an inspection of individual drug plasma concentration time profiles. The terminal elimination rate constant (*k*_*e*_) was determined by least-square regression analysis of terminal logarithm-linear portions of the plasma concentration time profile (*k*_*e*_ = −2.303 × slope). The elimination half-life (*t*_1/2_) was calculated as 0.693/*k*_*e*_. The AUC_0−*t*_ from time zero to the last quantifiable point (*C*_*t*_) was calculated using the trapezoidal rule, and extrapolated AUC from *C*_*t*_ to infinity (AUC_*t*−*∞*_) was determined as *C*_*t*_/*k*_*e*_. Total AUC_0−∞_ was the sum of AUC_0−*t*_ + AUC_*t*−*∞*_. 

#### 2.5.2. Statistical Analysis

Bioequivalence was evaluated by means of statistical analysis of variance (ANOVA) and Student's *t*-test for the crossover design with standard 90% confidence intervals (CI) of the test/reference ratio with logarithm-transformed data. The *T*_max⁡_ was analyzed by nonparametric test (Mann-Whitney test). The bioequivalence acceptance criteria required that the 90% CI for the test/reference ratios of the AUC and *C*_max⁡_ fell into 80%–125% for AUC_0−*t*_ and AUC_0−*∞*_ and 70%–143% for *C*_max⁡_, respectively [14, 15]. 

## 3. Results and Discussion

All 25 patients completed the study as per the protocol. Their mean values of age, weight, height, and body mass index were 30.9 ± 3.2 year, 75.8 ± 12.8 kg, 1.73 ± 0.10 m, and 25.46 ± 4.27 kg/m^2^, respectively. 

**Figure 1:**
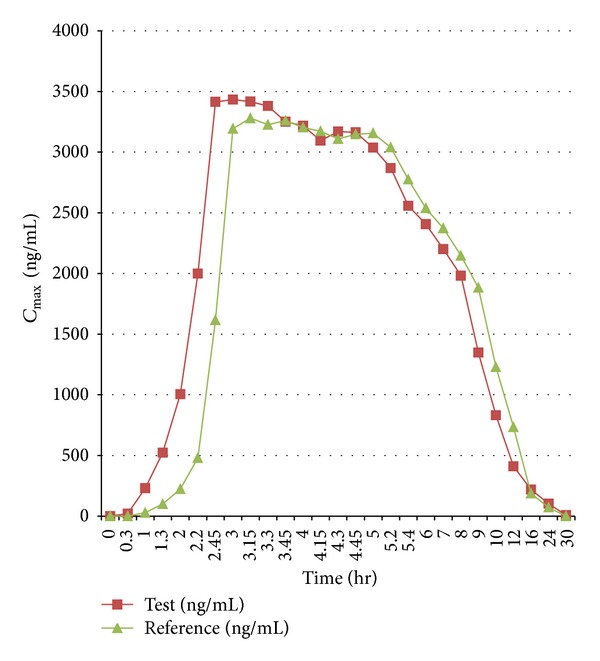
Mean ± SD plasma concentration time profiles after oral administration of 40 mg test formulation (Tripepsa) and reference (Pantocid) formulation under fasted condition.

Oral administrations of both 40 mg pantoprazole tablets were well tolerated. The mean plasma concentration time curves of test and reference were comparable (Figure 1). Taken together, all of the results mentioned above indicated that the two formulations have comparable pharmacokinetic profiles of pantoprazole. In the first two hours after the drug administration, *C*_max⁡_ of the test formulation was seen greater than the reference formulation. Moreover, fairly rapid absorption of pantoprazole from the test formulation in the intestine showed linear increase in the *C*_max⁡_ within 2.56 hr. This might be suspected due to the increased solubility and absorption of pantoprazole by HPBCD. It has been previously reported that HPBCD increased the apparent solubility of pantoprazole by 36 times [13]. 

The main pharmacokinetic parameters for test and reference formulations are listed in Table 1. The average half-life of test pantoprazole in serum (1.06–9.40, mean = 4.09 hr) was lower than the reference pantoprazole (2.06–11.20, mean = 5.38 hr); however, it was longer than the expected values reported from a previous study (1.25 hr) [3]. 

The mean values (±SD) of the *C*_max⁡_ and AUC_0−∞_ for test formulation were not significantly different from those of reference formulation (4057.04 ± 914.97 versus 3708 ± 720.75 ng/mL and 23907.75 ± 5745.31 versus 26369.31 ± 5965.38 ng·hr/mL). Bioequivalence analysis showed that 90% CI values for the test/reference ratios (%) of AUC_0−∞_ and *C*_max⁡_ were 90.21 (83.69–97.24) and 108.68 (100.21–117.86), respectively (Table 2). The coefficient of variation (%CV) estimated from S^2^ obtained from the ANOVA after logarithmic transformed for AUC_0−*∞*_ and *C*_max⁡_ was 24.03% and 22.62%, respectively. According to the nomograms and tables of Diletti, the power of tests values for AUC and *C*_max⁡_ were >90% and 80% for the sample size of 25, respectively. In addition, since the 90% CI values of AUC_0−*∞*_ and *C*_max⁡_ were within the bioequivalence range, our study demonstrated the bioequivalence of the two preparations. 

Based on the aforementioned results, the test formulation of pantoprazole sodium tablets (Tripepsa), formulated by Akums Drugs & Pharmaceuticals Limited, India, is considered bioequivalent with commercially available pantoprazole. 

## 4. Conclusions

The present randomized, two-way crossover design study indicated that two brands of pantoprazole sodium 40 mg preparations were bioequivalent. Hence, Tripepsa may have excellent therapeutic efficacy in patients with peptic acid disorders.

## Figures and Tables

**Table 1:** Comparison of pantoprazole pharmacokinetic parameters after single oral dose of 40 mg reference product (Tripepsa) and test product (Pantocid).

Pharmacokinetic parameter	Test product (Tripepsa)	Reference product (Pantocid)
*C*_ max_ (ng/mL)	4057.04 ± 914.97	3708 ± 720.75
*T*_ max_ (hr)	2.56 ± 0.33	3.36 ± 0.44
AUC_0−*∞*_ (ng·hr/mL)	23907.75 ± 5745.31	26369.31 ± 5965.38
AUC_0−*t*_ (ng·hr/mL)	23618.02 ± 5745.31	24351.05 ± 5965.38
AUC_0−*∞*_/AUC_0−*t*_ (%)	98.72 ± 0.28	92.03 ± 1.49
*t*_ 1/2_ (hr)	4.15 ± 2.41	5.64 ± 2.59

Values are given as mean ± SD.

**Table 2:** The mean and 90% confidence intervals (CI) of pharmacokinetic parameters of the test product (Tripepsa) compared to the reference product (Pantocid).

Pharmacokinetic parameter	Mean ratio (%)	90% CI (%)	Bioequivalence limit (%)
Ratio of *C*_max_	108.68	100.21–117.86	80–125
Ratio of AUC_0−*∞*_	90.21	83.69–97.24	80–125
Ratio of AUC_0−*t*_	96.78	89.56–104.58	70–143
